# Dying, dependency and death: Exploring palliative care access gaps for children

**DOI:** 10.12688/wellcomeopenres.20803.1

**Published:** 2024-03-01

**Authors:** Christina Lamb

**Affiliations:** 1 Faculty of Health Disciplines, Athabasca University, Athabasca, Alberta, T9SJA3, Canada; 2 CCBI, St. Michael's College, University of Toronto, Toronto, Ontario, M5S1J4, Canada

**Keywords:** Pediatric palliative care, dying, death, bioethics, dependency, population health, global health

## Abstract

Death has become a medicalized event. As such, end-of-life care has become entrenched in an over-reliance on individual patient autonomy to guide ethical decision making. Subsequently, the process of dying and the event of death are not primarily valued as life events – that is, as life-affirming phases of living. Rather, dying and death are viewed through the lens of medical options of when and how to die versus why dying and death are meaningful. This presents a problem for addressing the pediatric palliative care access gap and the Global Common Good. Specifically, in the context of important life events, one of which is death, we need space to be dependent on our inter-personal relationships to make crucial life decisions that affect our well-being. Recognizing dependency and inter-personalism is particularly important for pediatric populations. Children are uniquely placed to draw on their families and caregivers to make affirming life decisions in end-of-life care. This is particularly challenging to do in the Canadian context when Specialized Pediatric Palliative Care is not equitably available but options such as assisted death may soon be. Importantly, the meaning of death and dying is largely unexplored for this population. To advance ethical care at the end-of-life, more emphasis needs to be placed on the meaning that end of life events hold for Canadian children. In this paper I will outline the relevance of dependency and inter-personalism to attend to dying and death as meaningful phases of living for Canadian children and in relation to the pediatric palliative care access gap, the Global Common Good and Global Health Bioethics.

## Plain Language Summary

Around the world children need access to pediatric palliative healthcare during and at the end of life. In both Global Health and Canadian contexts, children have yet to achieve the necessary access to this kind of healthcare. At the same time, the meaning of dying and death is not well known in healthcare contexts, and this is particularly true for Canadian children. Instead, dying and death have become medicalized events in healthcare. This means that how and when to die get more consideration in healthcare than the meaning of dying and death as life events. This paper will look at how children can start to better access the care they need at the end of life, by discussing how dying and death should be considered as life events first and in relation to children. One way to think about this is to appreciate that children are physical, psycho-social, spiritual and dependent people who require inter-personal relationships to enact their care. Dying children should also be able to flourish for the Global Common Good. To start to address the pediatric palliative care access gap, the meaning of dying and death and palliative care need to become part of mainstream healthcare.

## Disclaimer

The views expressed in this article are those of the author(s). Publication in Wellcome Open Research does not imply endorsement by Wellcome.

## Introduction

Globally, there is a growing recognition that dying, and death have become medicalized events in healthcare ^1^. By medicalized event I mean that dying has come to be understood as an event that takes place in healthcare and that requires healthcare interventions in order for death to occur ^1^. However, the *meaning* of dying and death have not gained significant attention in healthcare contexts ^1^. Rather, the meaning of dying and death are often appreciated in terms of medical decision making around patient’s preferences about how they want to die, versus what dying, and death mean for them or what meaning these life events hold for them. Instead, dying and death ought to be viewed first as life events, and, when applicable, as life events that may benefit from healthcare as a supportive measure, and not as an exclusively interventionist approach.

While practical decisions about dying and death in the context of healthcare are important, more holistic approaches to care are also needed that appreciate what dying and death mean to the people experiencing them. Specifically, approaches that take into consideration the physical, psycho-social, and spiritual aspects of being human ^2^. Moreover, the failure to attribute proper meaning to dying and death in healthcare is an ethical problem because death is a significant life event. How we die and how death occurs, in other words, is laden with ethical content and yet the matter of how to experience this life event well in healthcare has been afforded far less attention than it deserves ^3^. This lack of attention manifests itself in varied ways, including the reality that one of the most pressing health issues in the world today is the lack of access to palliative care at the end of life ^4^. Of particular concern is the lack of access to palliative care for children.

Palliative care is an integrated form of healthcare services that aims to improve the quality of life of individuals and their families who face health-related challenges due to life-threatening or limiting diseases by early identification and treatment of physical, psychological, social, and spiritual suffering related to illness and disease ^5^. Palliative care can be initiated at diagnosis and continue throughout one’s disease trajectory up to and including palliative care at the end of life ^5, 6^. Annual, global estimates indicate that 25.7 million people need access to palliative care at the end of life ^7^. Approximately 7 percent of those in need are children ^7^. Pediatric palliative care (PPC) is “the active total care of the child’s body, mind, and spirit, and also involves giving support to the family” ^8^. PPC therefore encompasses a holistic approach to population-specific pediatric healthcare from perinatal up to end-of-life phases of life and anytime in between ^6^. For instance, some children may receive PPC as Specialized Pediatric Palliative Care (SPPC). SPPC is care that involves interdisciplinary teams of healthcare professionals and social workers with the expertise to provide PPC in established settings and who specialize in the care of families with children who have complex care needs ^2, 6^.

Although the PPC access gap is more prevalent for children in low to middle income level countries (LMIC), access disparities also exist in high income countries such as Canada. Specifically, and even though Canada has optimal PPC access relative to other countries, Canadian children lack equitable access to SPPC ^7, 9^. Widespread lack of social and professional knowledge about PPC and SPPC remain substantive barriers to its uptake and implementation in both Canada and internationally ^7, 10^. However, additional barriers exist. Specifically, the way that healthcare is perceived qua healthcare needs an ontological shift to include care of the dying as an innate part of healthcare provision. Namely, moving from a conceptualization of healthcare as a solely life-giving, sustaining, and prolonging enterprise to one that fundamentally values dying as an equally important phase of living. Similarly, palliative care should become a regular part of healthcare as opposed to an under-accessed area of specialization. Given that every person will die, those who die in relation to illness and disease ought to have the same access to care as those who are living.

Yet, including PPC as an established part of healthcare will be a significant shift in how healthcare is currently perceived. This is due, in part, because healthcare for children requires a unique approach that formally values dependency and inter-personalism since children live in dependent, person-centered, and relational contexts ^5^. Namely, children and all infants need adult decision makers involved in their care ^11^. This relationality is especially true in Canada, where there is little understanding about the meaning that dying and death hold for children, their families, and caregivers in the context of Canadian healthcare ^2^. Further, some children in Canada may soon be eligible for assisted death which has important, ethical implications for pediatric population health given the lack of access to SPPC in this country.

In this paper, I will discuss how to start to address the PPC access gap in Canada and in Global Health contexts by showing how PPC requires a unique approach in relation to an ontological re-articulation of healthcare. To illustrate this perspective, I will provide an overview of the PPC gap in Global Health and then Canadian-specific contexts. Next, I will define and discuss how the concepts of inter-personalism, and dependency are necessary for generating ethically meaningful discussions about the meaning of pediatric dying and death. Such discussions are essential in PPC and especially so when a child needs PPC at the end of life. I will complement this discussion with a Global Health bioethics approach by relating it to the Global Common Good, where an appreciation for dying as a positive phase of living is essential for addressing the global PPC access gap in general and the lack of SPPC in Canada in particular. By addressing these points, I hope to highlight how care for dying children needs a unique solution and to lay the initial ground for a fruitful discussion in which to do so, that is both relevant to children in Canada and around the world.

## Global Pediatric Palliative Care Access Gap

Most children who need PPC live in low to middle income countries ^6, 7, 12^. However, barriers also exist to children receiving care across high income countries. Such barriers include the absence or irregular implementation of national PPC strategies, policies, or programs; limited numbers of healthcare professionals with adequate training and subsequent working knowledge of palliative care principles and practices, and inadequate fiscal support for programs or access to opioid medication for pain control ^6, 7^. Many of these macro-level barriers are rooted in a scarcity of funding to create, implement, and sustain palliative care infrastructure in the countries that need it. Regardless of income level, most countries, Canada included, need enhanced societal awareness about PPC and SPPC ^6, 10^.

To start to address these multifaceted barriers to access, the authors of the *Lancet Commission on Palliative Care and Pain Relief* proposed that a basic, universal healthcare package be made available as a minimum care requirement ^4^. Such efforts have been echoed by the World Health Assembly and the Worldwide Hospice Palliative Care Alliance ^7^. Some countries that have successfully implemented PPC and PPC at the end of life more broadly into their national healthcare programs are also capacity building to operationalize their own pediatric palliative care programs ^7^. All efforts require recognition of cultural differences and experiences and the multifaceted challenges that each country as well as regional experiences to implement successful and sustainable programs.

In addition to the lack of resources and barriers to access that hinder practical solutions to the global PPC access problem, psycho-social issues also bar children from accessing PPC at the end of life. Namely, dying and death are not being valued in healthcare contexts ^1^. Various reasons exist for this, including an over-reliance on health systems to provide curative care when palliative care should be the focus ^13^. Discussion about dying and death has also receded across many societies, rooted in fear and avoidance of the reality of dying ^1^. Other reasons include the fact that people are not dying in the supportive presence of families or communities; lack of recognition that death is as much if not more of a spiritual and psychological than a physiological event, and a host of interconnected issues related to religion, race, ethnicity, gender, income as well as education which play complex roles in the lack of value that is being placed on dying and death around the world today ^1^. While many of these issues need to be parsed out further in relation to children’s unique dying and death experiences, the upshot is that the lack of attention being paid to the meaning of dying and death is a universal problem. Subsequently, research is needed to understand what dying and death mean to children ^2^.

Existing research shows that the search for this meaning is an under-explored phenomenon ^2, 14^. Relevantly, and based on my initial research on the meaning of dying and death for Canadian children, I will focus on the experience of children in Canada. Although PPC is needed for children in Global Health contexts and particularly in LMICs, focusing on the experience of children in Canada, a country that has achieved seemingly significant inroads into PPC, is relevant and helpful. Firstly, because each country has its own challenges, culture and contexts that need to be considered in relation to the universal need for PPC. Secondly, and despite its ample resources, Canada still lacks a national, palliative care infrastructure as well as equitable access to SPPC for children who need it. Moreover, the meaning of dying and death is relatively unknown for children in this country, which may impede continued progress in providing palliative care for pediatric populations in Canada.

## Canadian Pediatric Palliative Care Access Gap

The lack of equitable access to SPPC provision is especially troubling in Canada, where the majority of Canadian children with chronic, complex and/or life-limiting illnesses who need SPPC care aren’t able to access it ^2, 9^. However, Canadian children in need of SPPC only make up a fraction of the overall population in a healthcare system operating on the principle of universal access. The lack of available SPPC at the end of life for a faction of vulnerable Canadian children is therefore a marked health inequity.

This access disparity is also indicative of the broader, Canadian disparity concerning the general lack of access to PPC. Although calls for improvement have been made - resulting in access to SPPC being quadrupled for Canadian children between 2002–2012 - approximately 81% of children who could benefit from SPPC services are not ^2, 9^. Barriers to access include broader issues related to a lack of awareness on what PPC is, and subsequent and late referrals which parallel some of the global barriers to PPC implementation ^10^.

As well, PPC stakeholders have identified infant to adolescent and Indigenous children in Canada as under-serviced populations regarding their lack of access to palliative care. Yet, few national efforts have been made to enact change to address it ^15^. Further, assisted death may soon be legally available for mature minors in Canada ^10, 16^. Where assisted death is legally available in Canada, current guidelines stipulate that information about all other care options must be presented to patients requesting assisted death ^17^. While the extensive ethical, philosophical, and psychological challenges associated with this impending legislation are beyond the scope of this paper to address, these challenges prompt the question in relation to the care of dying Canadian children: without adequate access to PPC and SPPC in this country, what care options will mature minors have to choose from if they choose assisted death? ^18^

Two initial responses to this question are helpful. Firstly, this question highlights the significant need for PPC to be valued not only in Canadian healthcare, but in socio-political contexts as well. Since decisions about healthcare are rooted in social values which are especially impactful in countries with socialized healthcare systems, the need to value dying and death as meaningful phases of life is becoming a growing area of concern for Canadians in general, in relation to the PPC access gap, and given their rapid implementation of assisted death ^19, 20^. Because social values also predicate how dying and death need to be appreciated as life events, pediatric-specific bioethics approaches are needed in Canada to uncover better care options for children.

PPC therefore needs to become an inherent part of Canadian healthcare predicated on the social valuing of dying and death as meaningful events in the lives of children. As physical, psycho-social, and spiritual persons, children need to be able to make meaning of dying and death as it happens to them and then in the context of healthcare. In so doing, children require unique approaches to healthcare. Specifically, children require care that takes into consideration their dependency which is highlighted in interpersonal relationality. To elucidate these concepts, I will show how dependency and inter-personalism complement the overarching and fundamental need to value dying and death in healthcare as life events over medicalized events, and the relevance of this values shift in addressing the PPC access gap.

## Dependency and Inter-Personalism

Palliative care models of healthcare explicitly promote the fact that death is a natural part of life ^5, 7^. Unlike other approaches to mainstream healthcare (which focus predominantly on retaining or achieving independence in the context of illness), palliative care is an approach to health that takes into consideration that illness, dying, and death are a natural part of life. Yet, accompaniment necessitates inter-personal relationships. In adult care, these relationships ought to revolve around the concept of interdependency. For children, inter-personalism and accompaniment are necessarily dependent. For instance, children and all infants rely on adult decision-makers to enact their care ^11^. However, interpersonal relationality, interdependency, and dependency have yet to gain traction in healthcare as viable concepts in and of themselves, and in relation to autonomy-forward approaches that dominate bioethics approaches to medical decision making ^21^. Subsequently, achieving or sustaining autonomous positionality in medical decision making is not realistic for dying or ill people. And it is not the reality for children.

Rather, dependency is an inherent feature of being human. Nobody, adult, or child alike, can function wholesomely in the world without some degree of support ^21^. Whether this support manifests as knowledge, information, technology, people, or healthcare, being interdependent is a fact of life ^21^. Despite being foundational to the human experience, dependency is not explicitly featured in most definitions of healthcare ^21^. Consequently, adapting to the notion that palliative care should be part of mainstream healthcare requires a paradigmatic shift. This shift involves moving away from an over-reliance on the bioethical principle of autonomy, so that healthcare is not viewed as primarily aiming to achieve or sustain autonomy and independence in the context of illness.

By acknowledging that over-emphasizing autonomy and independence is, in fact, not appropriate in the context of illness or otherwise, we may start to appreciate that dying and death are not the medicalized loss of independence and autonomy. Rather, they are pivotal life moments in which one may need varying degrees of supportive healthcare or medical interventions. Such support and interventions, however, should not aim to minimize dying and death by over-medicalizing them. For instance, the medicalization of death and the parallel lack of value being placed on dying and death as normal, life events is a growing concern in Canada. In this country, end of life care is heavily reliant on the bioethical principles of autonomy and efficiency to drive medical decisions at the end of life ^20^.

Relevantly, most requests for assisted death in Canada are made based on the “loss of ability to engage in meaningful life activities” ^22^. Yet, dying from illness or life-limiting conditions will necessarily result in a loss of independency and some autonomous ability. As Beaudry points out, work needs to be done in Canada to promote the lives of the dying as those who are still living, versus medically expediting death as an ableist solution to a loss of autonomy ^20, 23^.

Recognizing that dependency is a necessary part of being human therefore prompts the need for healthcare interventions that shift values away from an over-reliance on autonomy to the person-centered notion of dependency, which is relationally required for children. For access to PPC and SPPC to advance in the world and in Canada, respectively, an appreciation for pediatric healthcare in the context of dependency and inter-personalism is essential to attend to the priorities and needs of the pediatric patients who require it. Locating this need in relation to the Global Common Good is ethically relevant for further ensuring that the PPC access gap is addressed.

## Implications for the Global Common Good

Attending to an ontological re-orientation of healthcare for children that explicitly values dependency and inter-personalism is essential for the Global Common Good. The Global Common Good is a set of conditions that need to be met so that all members of the global human community can attain objectives that make sense for everyone to achieve to thrive ^24^. Considering this principle, everyone should have the opportunity to flourish in life ^25^. This is no less true for those who are dying. As such, dying children should be afforded every opportunity to access healthcare requisite to their care needs. This access must be predicated on the fact that dying and death are life events that all people share and cannot be excluded from considerations around human flourishing. I argue this contingency is imperative for shifting the meta-paradigm of healthcare from being centrally oriented to caring for the living so that they recover from an illness or disease to an orientation that care should be provided for all those who are *living*, up to and including death as the final moment of life.

To appreciate such experiences requires caring for people holistically, and significant attention needs to be paid in healthcare to the meaning that dying, and death first hold for those who are experiencing them as life events, and then in the context of healthcare. Although it is not within the scope of this paper to provide an in-depth philosophical discussion of the dominant, ethical frameworks in relation to addressing the PPC access gap, it is worth noting that efforts to address this gap may fall short if they fail to include an explicit appreciation for children by way of their dependency and inter-personal relationships. These concepts are essential to children’s experiences, and subsequently to their experiences of the Common Good. For instance, although the *Lancet Commission on Death and Dying* provides recommendations to resurrect the meaning of death as a positive aspect of living, the ethical foundations underpinning their considerations require further clarification to sufficiently address palliative care disparities – pediatric or otherwise – in Canada and around the world ^1^. Specifically, without a clearer delineation of the importance and meaning that dying and death hold, the current lack of value placed on life in its final phases which underscores the PPC gap will remain unchanged.

Fulfilling the mandate of the Commission therefore requires a change in perspective, one which shifts care away from an over-reliant focus on autonomy in healthcare and towards the person-centered notion of dependency. To move forward in valuing death in pediatric, Global Health and palliative care contexts, value needs to be placed on dependency for children to access the Global Common Good. While this approach will not resolve all the barriers to expanding SPPC and other forms of palliative care in Canada or the Global Health context, re-valuing death needs to start with acknowledging the vulnerability of dependent life across mainstream healthcare. Subsequently, the further need exists to ensure that addressing the PPC access gap includes bioethical frameworks and policies that are pediatric-specific and aimed at providing care that accompanies children in their dying and death experiences. Research into this need is an important, next step in addressing the underlying philosophies needed to guide efforts to address the PPC gap.

## Conclusion

To start to address the PPC access gap in Canada and Global Health contexts, value needs to be placed on giving dying and death their proper place in life as well as in the context of healthcare. This valuing requires a paradigmatic shift in healthcare. Namely, by ensuring that palliative care is viewed as part of mainstream healthcare that supports and enhances dying and death as life events and simultaneously avoids, as the *Lancet* authors on the *Commission of Dying and Death* point out, unilaterally mechanizing healthcare into dying and death experiences. Rather, moving forward will require embracing the pediatric- specific concepts of inter-personalism and dependency which are necessary to appreciate what dying and death are to children and what supportive healthcare will mean to them. Ensuring that palliative care is part of mainstream healthcare offers a unique opportunity to support the lives of those who are dying as an opportunity for human flourishing. Research into the meaning of dying and death as well as the holistic relevance these life phases hold for children needs to be conducted to ensure common opportunities so that children, across every phase of life, have the freedom and support they need to flourish. In so doing, progress can be made to address the PPC and SPPC access gaps in global and Canadian contexts for children.

## Data Availability

No data are associated with this article.
