# Supplementary material for: Dying, dependency and death: Exploring palliative care access gaps for children
Source: Wellcome Open Res. 2024 Mar 1;9:120. [Version 1] doi: 10.12688/wellcomeopenres.20803.1 (PMC11109588; doi:10.12688/wellcomeopenres.20803.1)
Supplement: Peer review history

# Reviewer report: Reviewer response for version 1

**DOI:** 10.21956/wellcomeopenres.23021.r81469

**Published:** 2024-05-22

**Authors:** Jay R. Malone

**Affiliations:** 1 Washington University in St Louis, St. Louis, Missouri, USA

This article discusses the growing recognition that death and dying have become overly medicalized in healthcare, emphasizing the need to see them as significant life events rather than solely medical issues. It highlights the importance of holistic approaches to palliative care that consider physical, psycho-social, and spiritual aspects, particularly for children. The lack of access to pediatric palliative care (PPC), especially in low and middle-income countries, and even in high-income countries like Canada, is noted as a critical issue. The article calls for a shift in healthcare to better integrate PPC into mainstream healthcare, and to value dying as a natural part of life. This letter is well-written and highlights an important need in pediatric healthcare. 

 One possible critique is a need to address the existing work that cautions the field of palliative care over too vigorously integrating into mainstream healthcare (see, for instance: The Anticipatory Corpse, by Jeffrey Bishop). There is a tautological problem: medicine is overly technological and efficiency-focused. Palliative care desires to address death and dying holistically, and thus integrates itself into medicine. Palliative care, now a part of medicine, itself becomes overly technological and efficiency-focused, diminishing its ability to address death and dying holistically. 

 I agree with the author that death has become medicalized and that addressing the need for meaning-making within the now-medicalized context of death is an important task. Palliative care must be careful what it wishes for in entering into mainstream medicine, lest it be leveraged as one more efficiency-driven tool. Nearly the entirety of the author's argument supports this idea, but the conclusion calls for "ensuring that palliative care is viewed as part of mainstream healthcare". The author could strengthen the letter overall by at least acknowledging that the inclusion of palliative care in mainstream medicine may be a poison apple. Alternatively, the conclusion could be altered slightly to change the call for palliative care to become a part of mainstream healthcare, or to clarify that what is really meant here is simply that people, particularly children, ought to have access to a mechanism to holistically attend to needs related to death and dying.

Does the article adequately reference differing views and opinions?

Partly

Are all factual statements correct, and are statements and arguments made adequately supported by citations?

Yes

Is the Open Letter written in accessible language?

Yes

Where applicable, are recommendations and next steps explained clearly for others to follow?

Yes

Is the rationale for the Open Letter provided in sufficient detail?

Yes

Reviewer Expertise:

Health care ethics, pediatric ethics, end-of-life ethics

I confirm that I have read this submission and believe that I have an appropriate level of expertise to confirm that it is of an acceptable scientific standard, however I have significant reservations, as outlined above.

# Reviewer report: Reviewer response for version 1

**DOI:** 10.21956/wellcomeopenres.23021.r79132

**Published:** 2024-05-16

**Authors:** Jonathan Herring

**Affiliations:** 1 University of Oxford John Fell Fund, Oxford, England, UK

At the heart of this article is a call to look beyond autonomy in debates around end of life decisions concerning children.  The article powerfully calls for a discussion of seeking to understand the meaning of death and dying for children and how these can be experienced and understood as life events.  As the author suggests asking such questions is likely to draw out the importance of a relational understanding of death and nature of human dependency.  However, asking these questions also raises the problems of what the author describes as the "pediatric palliative care access gap" in the Canadian Context.  If death and dying are not simply "choice" or medical facts, but rather relational experiences and life events with particular meanings, the medical context (e.g. within palliative care) is required to enable children and those close to them to explore their meaning.  This is an important and valuable insight into the literature.

Does the article adequately reference differing views and opinions?

Yes

Are all factual statements correct, and are statements and arguments made adequately supported by citations?

Yes

Is the Open Letter written in accessible language?

Yes

Where applicable, are recommendations and next steps explained clearly for others to follow?

Yes

Is the rationale for the Open Letter provided in sufficient detail?

Yes

Reviewer Expertise:

Family law, medical law and ethics, law and caring.

I confirm that I have read this submission and believe that I have an appropriate level of expertise to confirm that it is of an acceptable scientific standard.

# Reviewer report: Reviewer response for version 1

**DOI:** 10.21956/wellcomeopenres.23021.r81474

**Published:** 2024-05-10

**Authors:** Natalie Bradford

**Affiliations:** 1 Queensland University of Technology, Brisbane, Queensland, Australia

Thankyou for the opportunity to review this thoughtful, and thought provoking open letter, that debates issues for pediatric palliative care and dying children in Canada.

 The author eloquently argues that more holistic approaches are needed to appreciate what dying and death mean to those affected and that the failure to do so has contributed to the lack of pediatric palliative care services across the globe.

 The important point is made that those who are dying should have the same access to care as those who are living. The pressing need for pediatric-specific bioethics approaches for the care options for dying children is well articulated.

 I have just a few comments and noted a couple of typos.

 1. In the introduction, the statement that dying is an event that takes place 'in healthcare'- I wonder if you mean *healthcare facilities*? or supported by *healthcare professionals*?

 2. Similarly - towards the end of that sentence- is it that interventions are considered necessary for a ' *good death'* to occur rather than death itself?

 3. There is a typo in 4th paragraph - remove ' *qua healthcare*'

 4. Second paragraph of section on access to PPC in Canada. The sentence ending in ' *approximately 81% of children who could benefit from SPPC services are not.*' is not quiet clear. Do you mean 81% who could benefit are not able to access? or not accessing PPC?

Does the article adequately reference differing views and opinions?

Yes

Are all factual statements correct, and are statements and arguments made adequately supported by citations?

Yes

Is the Open Letter written in accessible language?

Yes

Where applicable, are recommendations and next steps explained clearly for others to follow?

Yes

Is the rationale for the Open Letter provided in sufficient detail?

Yes

Reviewer Expertise:

Paediatric Palliative Care, Paediatric Cancer, Communication, Digital Health, Health Services Research

I confirm that I have read this submission and believe that I have an appropriate level of expertise to confirm that it is of an acceptable scientific standard.
